# Comparing Standard Keratometry and Total Keratometry Before and After Myopic Corneal Refractive Surgery With a Swept-Source OCT Biometer

**DOI:** 10.3389/fmed.2022.928027

**Published:** 2022-07-12

**Authors:** Ziyang Wang, Yanzheng Song, Wenli Yang, Dongjun Li, Wei Chen, Qi Zhao, Qian Liu, Changbin Zhai

**Affiliations:** Beijing Tongren Eye Center, Beijing Tongren Hospital, Capital Medical University, Beijing, China

**Keywords:** total keratometry, IOLMaster 700, corneal refractive surgery, cataract, IOL calculation

## Abstract

**Background:**

More recently, the swept-source OCT biometer-IOLMaster 700 has provided direct total corneal power measurement, named total keratometry. This study aims to evaluate whether standard keratometry (SK) and total keratometry (TK) with IOLMaster 700 can accurately reflect the corneal power changes induced by myopic corneal refractive surgery.

**Methods:**

In this study, the biometric data measured with the swept-source OCT biometer—IOLMaster 700 before and 3 months after the myopic corneal refractive surgery were recorded. The changes of biological parameters, including SK, posterior keratometry (PK), and TK, and the difference between SK and TK were compared. In addition, the changes of SK and TK induced by the surgery were compared with the changes of spherical equivalent at the corneal plane (ΔSEco).

**Results:**

A total of 74 eyes (74 patients) were included. The changes of SK, PK, TK, axial length, anterior chamber depth, and lens thickness after refractive surgery were all statistically significant (all *p* < 0.01), while the change of white-to-white was not (*p* = 0.075). The difference between SK and TK was −0.03 ± 0.10D before the corneal refractive surgery and increased to −0.78 ± 0.26D after surgery. The changes of SK and the changes of TK induced by the surgery had a good correlation with the changes of SEco (*r* = 0.97). ΔSK was significantly smaller than ΔSEco, with a difference of −0.65 ± 0.54D (*p* < 0.01). However, the difference between ΔTK and ΔSEco (0.10 ± 0.50D) was not statistically significant (*p* = 0.08).

**Conclusions:**

Using SK to reflect the changes induced by the myopic corneal refractive surgery may lead to underestimation, while TK could generate a more accurate result. The new parameter, TK, provided by the IOLMaster 700, appeared to provide an accurate, objective measure of corneal power that closely tracked the refractive change in corneal refractive surgery.

## Background

The calculation of intraocular lens (IOL) power after corneal refractive surgery has always been a popular and yet hard-to-tackle issue in cataract research, mainly for the following two reasons: the keratometry measurement error and the effective lens position (ELP) prediction error (1). The keratometry measurement error usually originates from: (1) the change of the ratio of the anterior to posterior corneal curvature radius (the A/P ratio) invalidates applicability of the standard keratometric index (1.3375); (2) it is difficult to obtain accurate anterior curvature with the standard keratometer or corneal topographer due to the substantial power change in the central cornea (2). The conventional measurement will lead to the overestimation of corneal power after refractive surgery, resulting in severe “hyperopia error” after cataract surgery (3, 4). Therefore, in order to achieve the accurate calculation of IOL power after corneal refractive surgery, it is necessary to revise the conventional simulated keratometry (SimK) or develop new instruments to directly measure total corneal power.

The IOLMaster 700 (Carl Zeiss Meditec AG) based on swept-source OCT (SS-OCT) technology shows great advantages in biometry. It can provide full-length OCT image to ensure the accuracy of axial measurement, and also offers telecentric keratometry, which is a distance-independent approach that allows repeatable measurements even for restless patients. Recently, IOLMaster 700 has introduced a new parameter, named total keratometry (TK), by combining telecentric keratectomy with SS-OCT to measure both anterior and posterior corneal surface simultaneously. It has been proved that the new IOLMaster 700 has good repeatability (5, 6), and the new parameter TK performs well in IOL power calculation (7–11). However, there is no evaluation on whether TK can accurately reflect the corneal power after refractive surgery. In this study, the changes of standard keratometry (SK) and TK before and after myopic corneal refractive surgery were recorded and compared with the change of spherical equivalent on the corneal plane (ΔSEco) induced by the surgery.

## Methods

### Patients

This prospective cross-sectional study comprised patients who underwent myopic corneal refractive surgery, including femtosecond laser-assisted *in situ* keratomileusis (LASIK) and small incision lenticule extraction (SMILE) in the refractive center in December 2020. All the corneal refractive surgeries were performed by the same physician (ZCB). Exclusion criteria were: (1) patients with other ocular diseases except ametropia, including corneal diseases, dry eyes, cataract, glaucoma, and fundus diseases; (2) patients with the previous history of ocular trauma and surgery; (3) patients with surgical complications.

The study adhered to the tenets of the declaration of Helsinki and was approved by the ethics committee of Beijing Tongren Hospital, Capital Medical University (TRECKY2018-049), and informed consent was obtained from all the patients.

### Instrument

Based on SS-OCT technology, the IOLMaster 700 (software version 1.70) uses a 1,055-nm wavelength swept-source technology to obtain the length parameters on the optic axis, including central corneal thickness, anterior chamber depth (ACD), lens thickness (LT), and axial length (AL). Iris recognition system is used to measure the horizontal corneal white-to-white diameter (WTW). The following two types of corneal power could be provided:

SK: The SK is calculated based on the anterior corneal curvature from measuring reflections of 18 light-emitting diodes, combined with telecentric keratometry. The standard keratometric index 1.3375 was used to convert the corneal curvature values in millimeters to values in diopters (D).

TK: The IOLMaster 700 first builds a toric anterior surface model from the telecentric 3-zone K and then measures pachymetry using SS-OCT in the six meridians. The pachymetry values are fitted to the anterior surface model to create the toric posterior surface model. The TK is calculated from the anterior and posterior corneal curvatures, as well as corneal thickness, by means of a thick lens formula.

### Measurements

Measurement by the IOLMaster 700 was performed for all the patients before and 3 months after the refractive surgery, and all the measurements were carried out by the same operator (WZY) under the same natural light without cycloplegia. Measure three times consecutively, and the average value of SK, TK and posterior keratometry (PK) was recorded. Mean keratometry was defined as the average of steep and flat keratometries, abbreviated as SKm, TKm, and PKm.

The subjective manifest refraction was also performed subsequently, and then converted to a spherical equivalent corneal plane value using a vertex distance of 12 mm. Delta SEco induced by corneal refractive surgery was used as the reference for evaluating different corneal power measurements (12).

### Statistical Analysis

SPSS 22.0 and MedCalc20.0 were used for the statistical analysis. The Kolmogorov Smirnov test was carried out to test the normality of the data, and the measurement results were described as mean ± SD. Comparison of the same parameters before and after the surgery, SK and TK, and SEco were all conducted with paired *t*-test. Bland Altman analysis was adopted to evaluate the agreement between the parameters, and the 95% limits of agreement (LoA) were calculated using the mean difference of ±1.96 SD. Narrower LoA means better agreement. The Pearson correlation coefficient *r* was calculated to evaluate the correlations between parameters and an *r* of >0.6 was considered to indicate a high correlation. A scattergram was drawn to perform a linear regression analysis. *p* < 0.05 was considered statistically significant.

## Results

A total of 74 patients who underwent corneal refractive surgery with an average age of 27.38 ± 6.00 years (ranged from 18 to 46) were enrolled, including 18 males and 56 females. Fifty-one patients underwent SMILE, and 23 patients underwent LASIK. The manifest refraction spherical equivalent was −6.43 ± 2.02D before surgery and 0.25 ± 0.54D after surgery. For all the patients, only the data on the right eye were included.

### Biometric Parameters

Table 1 shows both SK and TK decreased after myopic corneal refractive surgery. The differences were −5.78D for steep SK, −5.37D for flat SK, −5.58D for mean SK (SKm), −6.53 for steep TK, −6.12D for flat TK, and −6.33D for mean TK (TKm), and all differences were statistically significant (*p* < 0.01). The posterior cornea surface became slightly flatter after surgery, and the differences were 0.04D for steep posterior keratometry (PK), 0.02D for flat PK, 0.03D for mean PK. Both AL and ACD decreased, while the LT increased, and all changes were statistically significant (*p* < 0.01). However, WTW was unchanged (*p* = 0.075).

**Table 1:** Biometric parameters before and after myopic corneal refractive surgery by IOLMaster 700.

**Parameter**	**Before surgery**		**After surgery**		**Difference**	**t value**	***P*** **-value**
	**Mean ±SD**	**Total range**	**Mean ±SD**	**Total range**			
SKs (D)	44.36 ± 1.33	41.16, 46.78	38.57 ± 2.06	33.56, 42.78	−5.78 ± 1.81	−27.49	0.000
SKf (D)	43.19 ± 1.25	40.77, 46.62	37.83 ± 2.00	32.95, 42.26	−5.37 ± 1.81	−25.56	0.000
SKm (D)	43.78 ± 1.24	40.97, 46.70	38.20 ± 2.02	33.42, 42.44	−5.58 ± 1.78	−26.91	0.000
PKs (D)	−6.07 ± 0.23	−6.58, −5.57	−6.03 ± 0.22	−6.60, −5.52	0.04 ± 0.06	6.01	0.000
PKf (D)	−5.77 ± 0.20	−6.24, −5.38	−5.75 ± 0.20	−6.23, −5.32	0.02 ± 0.07	2.65	0.010
PKm (D)	−5.92 ± 0.21	−6.37, −5.50	−5.89 ± 0.20	−6.41, −5.44	0.03 ± 0.06	4.78	0.000
TKs (D)	44.26 ± 1.30	41.19, 46.68	37.73 ± 2.24	31.89, 42.17	−6.53 ± 2.06	−27.30	0.000
TKf (D)	43.22 ± 1.25	40.67, 46.68	37.10 ± 2.20	31.76, 41.96	−6.12 ± 2.07	−25.45	0.000
TKm (D)	43.74 ± 1.22	40.93, 46.68	37.42 ± 2.21	31.78, 42.07	−6.33 ± 2.03	−26.70	0.000
AL (mm)	25.79 ± 0.91	23.25, 28.33	25.68 ± 0.90	23.15, 28.12	−0.12 ± 0.04	−24.35	0.000
ACD (mm)	3.68 ± 0.25	2.93, 4.27	3.48 ± 0.24	2.77, 4.02	−0.21 ± 0.08	−22.00	0.000
LT (mm)	3.69 ± 0.28	3.17, 4.54	3.75 ± 0.27	3.19, 4.56	0.06 ± 0.05	10.28	0.000
WTW (mm)	11.99 ± 0.44	10.82, 12.92	11.95 ± 0.43	11.17, 12.94	−0.03 ± 0.16	−1.81	0.075

*SKs, steep standard keratometry; SKf, flat standard keratometry; SKm, mean standard keratometry; PKs, steep posterior keratometry; PKf, flat posterior keratometry; PKm, mean posterior keratometry; TKs, steep total keratometry; TKf, flat total keratometry; TKm, mean total keratometry; AL, axial length; ACD, anterior chamber depth; LT, lens thickness; WTW, white-to-white*.

### Comparison of SK and TK

**Figure 1:**
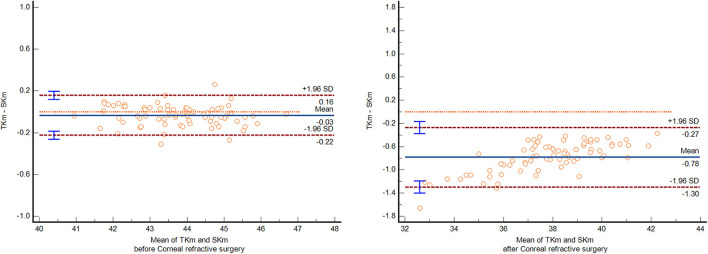
The Bland Altman plot of SK and TK before and after myopic corneal refractive surgery.

As Table 2 shows, before refractive surgery, the difference between SKm and TKm was −0.03 ± 0.10D, and 95% LoA was (−0.22, 0.16) D. After surgery, the difference increased. TKm was significantly smaller than SKm, and the difference was −0.78 ± 0.26D, with a 95% LoA of (−1.30, −0.27) D (*p* < 0.01). In addition, after surgery, the flatter the cornea was, the greater the difference was, as in Figure 1.

**Table 2:** The differences and agreements between SK and TK.

	**SKm(D)**	**TKm(D)**	**Difference**	**t value**	***P*** **-value**	**95% LoA**	
						**Lower**	**Upper**
Before surgery	43.78 ± 1.24	43.74 ± 1.22	−0.03 ± 0.10	−2.90	0.005	−0.22	0.16
After surgery	38.20 ± 2.02	37.42 ± 2.21	−0.78 ± 0.26	−25.70	0.000	−1.30	−0.27

*SKm, mean Standard Keratometry; TKm, mean total keratometry*.

### Comparison of the Changes of SK and TK With the Changes of SEco

**Figure 2:**
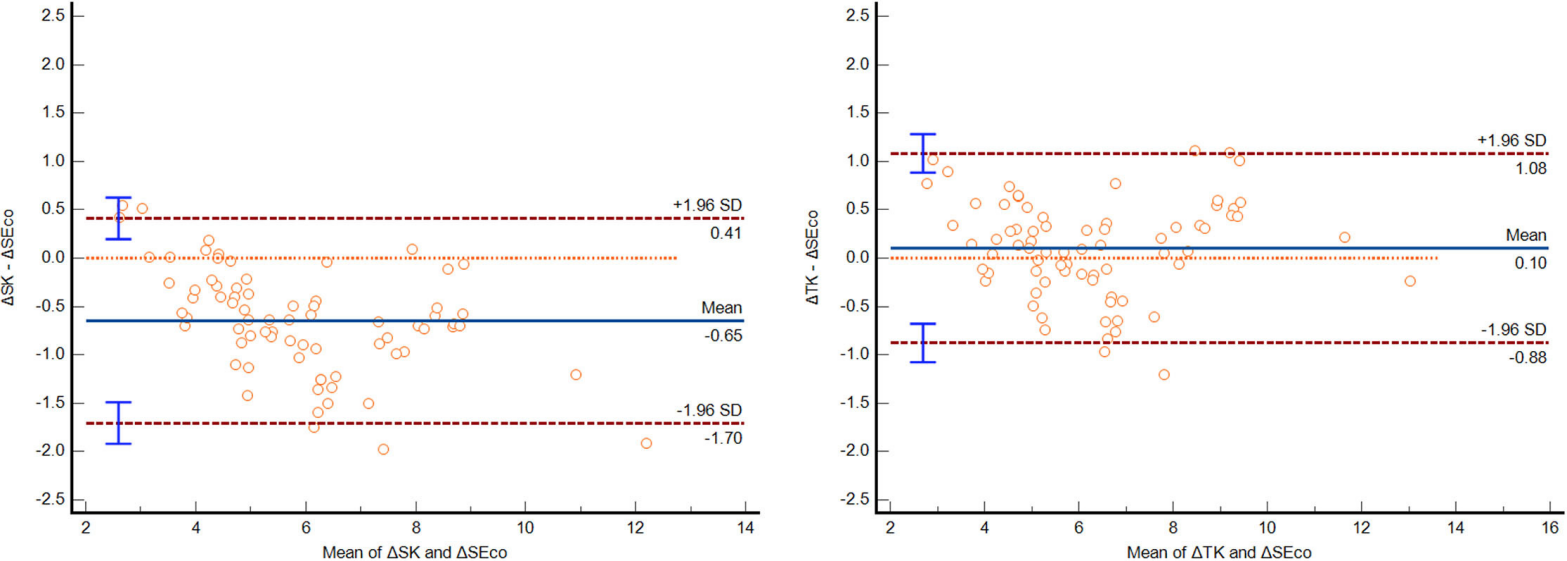
The Bland Altman plot of ΔSEco and ΔSK, and ΔSEco and ΔTK.

**Figure 3:**
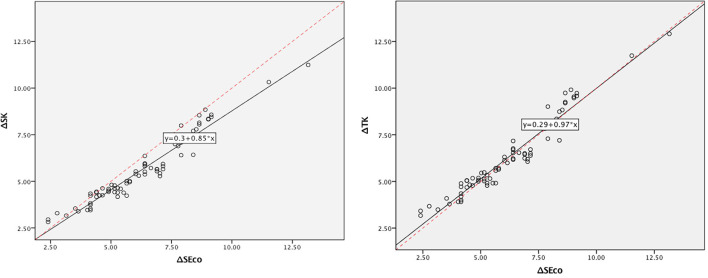
Linear regression analysis on ΔSEco and ΔSK, and ΔSEco and ΔTK.

The ΔSK and ΔTK induced by corneal refractive surgery were highly correlated with the ΔSEco, with a correlation coefficient of 0.97. The ΔSK (5.58 ± 1.78D) was significantly smaller than the ΔSEco (6.22 ± 2.04D), and the difference was −0.65 ± 0.54D, with a 95% LoA of (−1.70, 0.41) D (*p* < 0.01). The greater the amount of myopic correction was, the greater the difference was. However, the difference between ΔTK (6.33 ± 2.04D) and ΔSEco was only 0.10 ± 0.50D (*p* = 0.08), with a 95% LoA of (−0.88, 1.08) D (Table 3). Bland Altman plot and Linear regression analysis are shown in Figures 2, 3.

**Table 3:** Comparison of ΔSEco, ΔSK, and ΔTK before and after myopic corneal refractive surgery.

**Parameter**		**Difference with ΔSEco**	**t value**	***P*** **-value**	**95% LoA**		**Pearson correlation**	
					**Lower**	**Upper**	**r-value**	***P*** **-value**
ΔSEco	6.22 ± 2.04							
ΔSK	5.58 ± 1.78	−0.65 ± 0.54	−10.31	<0.01	−1.70	0.41	0.97	<0.01
ΔTK	6.33 ± 2.04	0.10 ± 0.50	1.78	0.08	−0.88	1.08	0.97	<0.01

*SEco, spherical equivalent on corneal plane; SK, standard keratometry; TK, total keratometry*.

## Discussion

Corneal refractive surgery changes the anterior surface of cornea, while the posterior surface is less affected. Therefore, the total corneal power cannot be reflected simply by the anterior surface curvature radius and an index of 1.3375, but both the anterior and posterior corneal data should be taken into consideration (13). The new IOLMaster 700 introduced a new parameter, TK. The anterior surface is obtained by telecentric keratometry, and then the SS-OCT technology is used to calculate the posterior surface data, thereby generating the total corneal curvature. This study aims to evaluate the new parameter TK can accurately reflect the corneal power after corneal refractive surgery.

In the current study, the changes of biometric parameters before and after surgery were recorded. Apart from the significant decrease in SK and TK, the study also found that the posterior surface slightly flattened, with a refractive power difference ranging from 0.02 to 0.04D. Ganesh et al. (14) used SCHWIND SIRIUS, which found that, after SMILE surgery, due to the changes of corneal biomechanics, the posterior cornea surface bulges forward under the action of intraocular pressure, and the refractive power of the posterior surface increases, which is contrary to our findings. We speculate that may be due to different measurement methods. The IOLMaster 700 provides posterior surface data using the SS-OCT technology based on the anterior surface measurement, rather than measuring directly. Therefore, the change of the anterior surface will cause a comparatively large impact on the posterior surface. Interestingly, the current study found that the LT increased by 0.06 ± 0.05 mm, and one possible explanation would be that the decrease of corneal refractive power led to the increase of lens accommodation when looking at the same visual target (15). The thinning of corneal thickness and the increase of LT resulted in the decrease of the ACD (including corneal thickness).

At present, many devices can measure total corneal power: true net power (TNP) with the Pentacam, real power (RP) with the CASIA, and TK with the IOLMaster 700, and all of them follow the Gaussian optics formula for thick lenses. The keratometry based on Gaussian optics formula allows the simultaneous measurement of the anterior and posterior corneal surfaces and uses the real refractive index to calculate the total power. The formula is K_GOF_ = (n_1_-n_0_)/R_anterior_ + (n_2_-n_1_)/R_posterior_ – (CCT/n_1_) × [(n_1_-n_0_)/R_anterior_] × [(n_2_-n_1_)/R_posterior_], where the refractive index of air is n_0_ = 1.000, the real corneal refractive index is n_1_ = 1.376, and the aqueous humor refractive index is n_2_ = 1.336. Previous studies have shown that K_GOF_ is smaller than SimK. In normal eyes, SimK is about 1.0–1.5D larger than K_GOF_ (16–19), and, after corneal refractive surgery, this difference will increase to 1.5–1.7D (20, 21), which may be caused by the following two factors: (1) Reference planes are different. The reference plane of SimK is the cornea posterior vertex, while K_GOF_ uses the second principal plane in front of the cornea, which could lead to a difference up to 0.8D; (2) The standard keratometric index 1.3375 used by SimK assumes the A/P ratio to be a fixed value, while, in reality, the A/P ratio is not absolutely constant. After refractive surgery, the A/P ratio deviates further from the normal range (22), and the difference caused by the second factor would further increase correspondingly. Therefore, in order to avoid the influence of simulated keratometric index, it is necessary to measure both the anterior and posterior corneal surfaces to obtain the total corneal power after refractive surgery.

The current study found that the difference between SKm and TKm was (−0.03 ± 0.10) D in unoperated eyes and (−0.78 ± 0.26) D after the refractive surgery, and both were significantly smaller than the previously reported mentioned above, which may be related to the internal revision of the instrument. According to the IOLMaster 700 manufacturer, TK has been revised to be closer to SK, and the new instrument allows the direct use of the currently available formula and IOL constantly provided by the ULIB website without optimization (23). However, the manufacturer did not publish the method for the revision. Previously, Srivannaboon and Chirapapaisan (7) has reported an A/P ratio of 1.13 (7.58/6.73 mm) among 60 patients with cataract with the IOLMaster 700. Similarly, in the current study, we calculated the A/P ratio of unoperated eyes was 1.14 (7.72/6.77 mm). Intriguingly, this ratio is very close to that of the Gullstrand model eye (1.13) (24). According to Haigis (25), the Gullstrand model can achieve equivalence to SimK by transforming the reference plane to the posterior surface. Therefore, we speculate that the IOLMaster 700 may have revised the reference plane to reduce the difference between TK and SK.

Previous studies have reported that total corneal power can better reflect the changes induced by corneal refractive surgery compared with SimK. For instance, Sónego-Krone et al. (26) found that manifest refraction change was best estimated by 4-mm total optical power from Orbscan; the mean difference was −0.08 ± 0.53 D and the correlation coefficient of 0.87. Tang (12) reported that the difference between the total power (3mm) provided by CAS-OCT and manifest refraction was −0.16 ± 0.44 D. In the current study, we found that the revised TK could also accurately reflect the changes of corneal power, and ΔTK is highly correlated with ΔSEco, with a correlation coefficient of 0.97 and a difference of only 0.10 ± 0.50D. The revised TK is appeared to provide a more accurate and objective measure of corneal power, which provides a theoretical basis for the good performance of TK in IOL calculation after corneal refractive surgery (7–11). However, the wide range of the LoA should be noticed, with a 95% LoA of (−0.88, 1.08) D. Hence, in some special cases, the prediction variance of TK should not be ignored.

One of the shortcomings of this study is including both SMILE and LASIK surgical methods. Some may argue that different surgical methods may affect the final results, which requires further research with data stratified. In addition, actual clinical result after cataract surgery of actual patients undergoing cataract surgery was lacking in the current study; therefore, the comparison of TK and SK has some certain limitations.

In conclusion, using SK to reflect the changes induced by the myopic corneal refractive surgery may lead to underestimation, while TK could generate a more accurate result. The new parameter, TK, provided by the IOLMaster 700, appeared to provide an accurate, objective measure of corneal power that closely tracked the refractive change in corneal refractive surgery.

## Data Availability Statement

The original contributions presented in the study are included in the article/supplementary material, further inquiries can be directed to the corresponding author/s.

## Ethics Statement

The studies involving human participants were reviewed and approved by the Ethics Committee of Beijing Tongren Hospital, Capital Medical University. The patients/participants provided their written informed consent to participate in this study.

## Author Contributions

ZW acquired and analyzed the data, drafted the initial manuscript, and revised the manuscript. YS, QL, DL, WC, and QZ collected data. WY and CZ conceptualized and designed the study, coordinated and supervised data collection, critically reviewed the manuscript, and revised the manuscript. All authors read and approved the final manuscript.

## Funding

This work was supported by the Hospital Founding of Beijing Tongren Hospital (Grant No. 2021-YJJ-PY-002).

## Conflict of Interest

The authors declare that the research was conducted in the absence of any commercial or financial relationships that could be construed as a potential conflict of interest.

## Publisher's Note

All claims expressed in this article are solely those of the authors and do not necessarily represent those of their affiliated organizations, or those of the publisher, the editors and the reviewers. Any product that may be evaluated in this article, or claim that may be made by its manufacturer, is not guaranteed or endorsed by the publisher.
